# Supplementary material for: Automatically annotated motion tracking identifies a distinct social behavioral profile following chronic social defeat stress
Source: Nat Commun. 2023 Jul 18;14:4319. doi: 10.1038/s41467-023-40040-3 (PMC10354203; doi:10.1038/s41467-023-40040-3)
Supplement: Supplementary file 1 — Supplementary Information [file 41467_2023_40040_MOESM1_ESM.pdf]

Automatically annotated motion tracking identifies a distinct social behavioral profile following chronic social defeat stress

Supplemental material

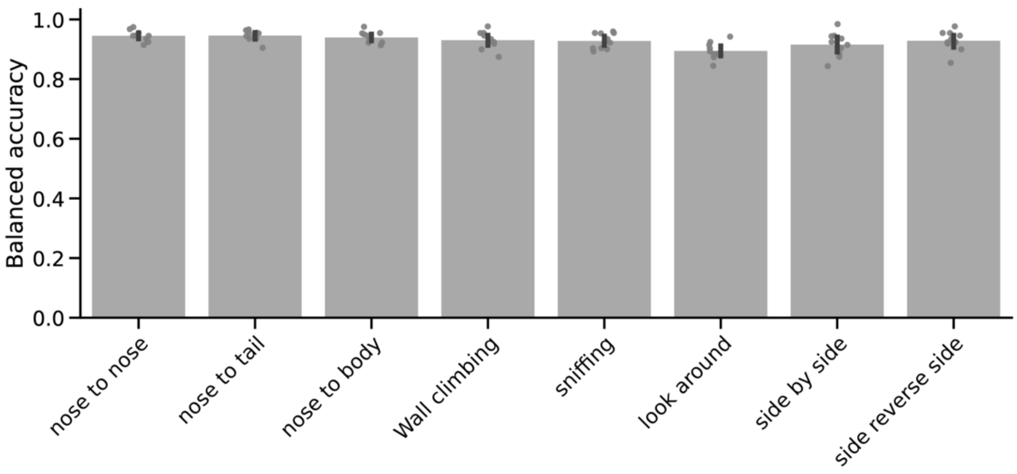

Supplemental figure 1. Validation of rule-based annotated behaviors. 10 out of 53 videos were manually labeled for all annotators (excluding stopped-and-huddled, see supplemental figure 2) using the Colabeler software (v2.0.4). Balanced accuracy between manual labels and predicted binary outcomes (presence or absence of a given trait at a given time) is reported**.** Bars represent the mean ± standard deviation across all 10 videos (N=10). Source data are provided as a Source Data file.

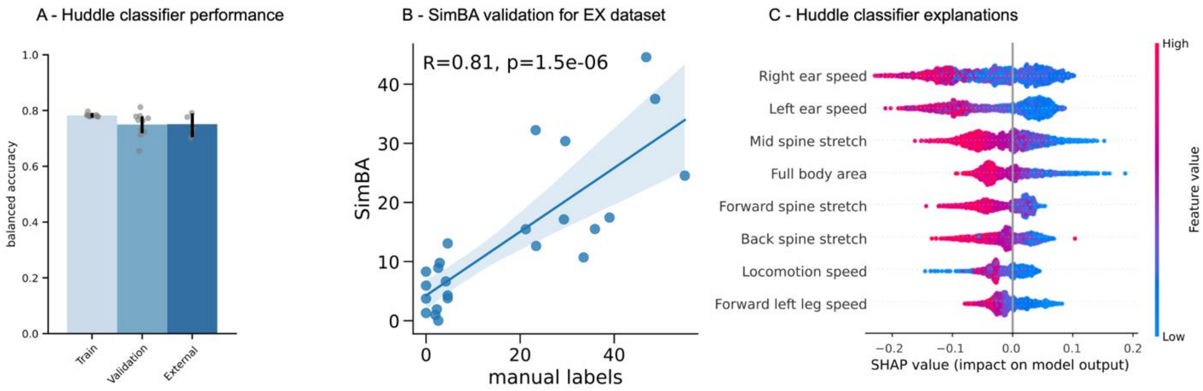

Supplemental figure 2. Validation of stopped-and-huddled classifier. A) Bar charts (mean ± standard deviation) showing balanced accuracy performance for the huddle classifier provided with the supervised pipeline within DeepOF. A total of 567367 video frames were either manually labeled (for the SI, OF, and SA datasets) or pseudo-labeled using SimBA (EX dataset) for the stopped-and-huddled trait using the labeling tool provided with SimBA v1.31.1. Labelling was conducted in four independent datasets (SI, OF, SA, and EX; see the animals' section in materials and methods for details), and two validation tasks were conducted, marked as "Validation" and "External" respectively. First, a 10-fold stratified cross-validation loop was executed within the SI dataset (which has the most labels, see supplemental table 2 for details), to test for overfitting and generalization within a single dataset. Balanced accuracy results were 0.78±0.005 and 0.75±0.046 for the training and validation sets, respectively (N=10). Second, a leave-one-dataset out cross-validation was conducted across all four datasets, to test whether the model can generalize to novel settings (different bedding, different arenas, different labs). A balanced accuracy of 0.75±0.04 was reported (N=4). B) SimBA validation of the classifiers used for pseudo-labelling in the external dataset. Correlation between total behavior duration (in seconds) in manual and predicted labels shown for all 24 videos (N=24). Both sets show a Pearson correlation coefficient ρ=0.81, which significantly deviates from zero (p-value=1.5e-6). Error bands represent the 95% confidence interval. C) SHAP analysis of the deployed model (trained in the whole dataset, with all concatenated four sites). The top 8 features are displayed of a total of 26 features including distances between body parts, speeds, and areas. Results show low head movement, low spine stretch, low body area, and low locomotion speed as the most important features for the model, which goes in line with the accepted definition of the behavior. Source data are provided as a Source Data file.

Supplemental table 1. Default thresholds used by the annotation pipeline in DeepOF

Annotated trait	Rule	Default threshold in DeepOF
**Nose-to-nose**	Nose to nose distance	< 25 mm
**Nose-to-tail**	Nose to tail distance	< 25 mm
**Nose-to-body**	Nose to any other body part	< 25 mm
**Side-by-side**	Nose to nose distance	< 45 mm
	Tail to tail distance	< 45 mm
**Side-reverse-side**	Nose to tail distance	< 45 mm
**Wall-climbing**	Nose reach beyond walls	> 10 mm
**Sniffing**	Nose distance to object	< 10 mm
	Nose speed	> 50 mm/s
	Locomotion speed	< 50 mm/s
**Look-around**	Locomotion speed	< 50 mm/s
	Nose speed	> 50 mm/s

Supplemental table 2. Datasets used in the current study

Dataset name	Experiment code	Number of videos	Frame rate	Video length	Labeled frames (stopped-and-huddled)	Prevalence (stopped-and-huddled)
Social interaction (SI)	1	53	25	10 min<br>15000 frames	299.350	10.83%
Open field (OF)	1	53	25	10 min<br>15000 frames	179.979	2.75%
Social avoidance (SA)	1	120	13	2.5 min<br>1950 frames	22.488	4.36%
Social interaction for SA resiliency (figure S6)	2	64	30	10 min<br>18000 frames	0	-
Social interaction (external)	3	20	30	1.5 min<br>2730 frames	65.550	14.95%
Total	-	310	-	-	567.367	8.49%

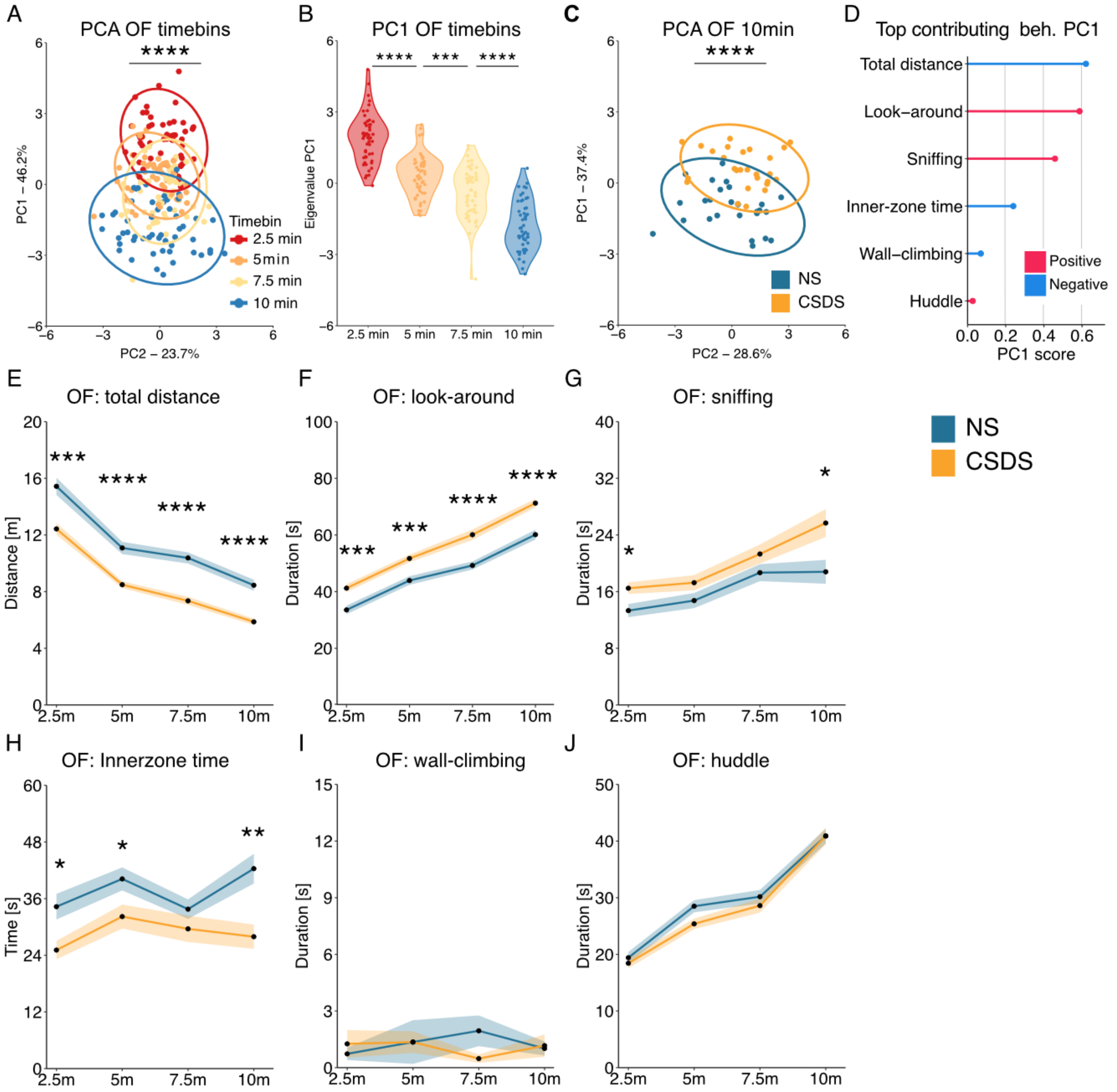

Supplemental figure 3. DeepOF behavioral classifiers in the open field task. A) The OF PCA time bins show a significant main effect (one-way ANOVA: F(3,208)=129.12, p=2.97e-47). B) Benjamini-hochberg (BH) posthoc shows that the time bins are significantly different from each other (2.5vs5, p=3.93e-14; 5vs7.5, p=0.0003, 7.5vs10, p=3.1e-12. C) The 10min OF PCA analysis shows a significant difference between conditions; independent samples *t*-test: T(51)=–7.23, p=2.37e-9. Data consisted of all the individual DeepOF behavioral classifiers, as listed in Figure 1C. D) The ranked behaviors on the PC1 using the corresponding rotated loading scores. E) The total distance was lower in CSDS animals; posthoc BH: 2.5 min T(51)=16.89, p=0.0001, 5 min T(51)=28.28, p=3.13e-6, 7.5 min (T(51)=39.59, p=2.86e-7, and 10 min (T(51)=33.77, p=8.1e-7). Two-way ANOVA on condition: F(1,208)=92.586, p=2.31e-18, time: F(1,208)=265.77, p=4.85e-39, condition×time: F(1,208)=0.10, p=0.75). F) Look-around was higher in CSDS

animals; posthoc BH: 2.5 min (T(51)=14.08, p=0.0004, 5 min (T(51)=14.84, p=0.0004), 7.5 min (T(51)=21.65, p=4.7e-5, and 10 min (T(51)=23.25, p=4.7e-5). Two-way ANOVA on condition: F(1,208)=74.04, p=1.9e-15, time: F(1,208)=356.65, p=5.4e-47, condition×time: F(1,208)=1.90, p=0.17). G) Sniffing was higher in CSDS animals for the 2.5- and 10 min time bins; posthoc Wilcoxon: W=199.5, p=0.023; W=210, p=0.023, respectively. The 5- and 7.5 min were not altered (W=258, p=0.13, and W=307, p=0.44, respectively). Kruskal-Wallis test 2.5 min: H(1)=7.27, p=0.024, 5 min: H(1)=2.74, p=0.13, 7.5 min: H(1)=0.6, p=0.43, and 10 min: H(1)=6.29, p=0.024. H) The inner zone time was lowered in CSDS animals for the 2.5, 5, and 10 min time bins; posthoc BH: T(51)=7.70, p=0.016, (T(51)=5.16, p=0.036, (T(51)=12.74, p=0.0032, respectively). The 7.5 min was not altered (p=0.24). Two-way ANOVA on condition: F(1,208)=24.04, p=1.9e-6, time: F(1,208)=2.07, p=0.15, condition×time: F(1,208)=0.53, p=0.47). I) Climbing did not reveal any difference using the Kruskal-Wallis test. J) Huddle did not reveal any difference using the Kruskal-Wallis test. The PCA graphs are provided with a 95% confidence ellipse and all individual samples as points. Further PC1 analyses are represented with a violin plot and all individual samples as points. The timeline graphs are presented as mean ± standard error of the mean. N=26 for NS and n=27 for CSDS in panels A-J. Source data are provided as a Source Data file.

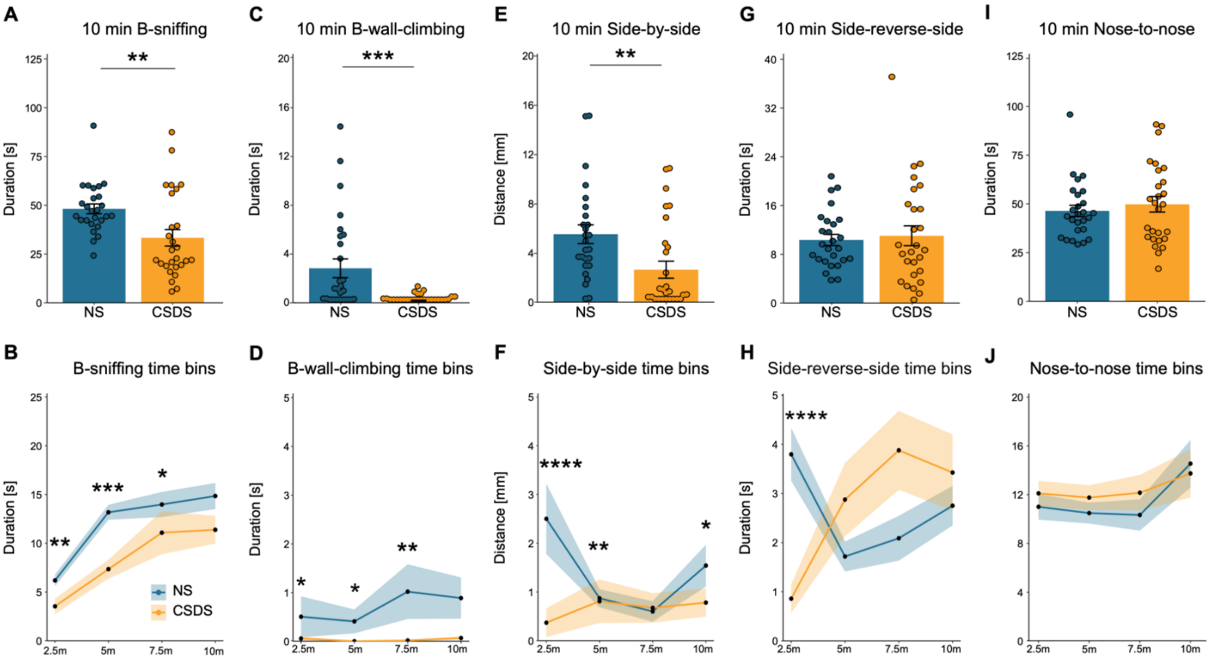

Supplemental Figure 4. DeepOF other behavioral classifiers in the social interaction task for 10 min duration. A) B-sniffing is lower in CSDS animals. Independent samples *t*-test: T(51)=2.99, p=0.004. B) Wilcoxon posthoc analysis revealed that B-sniffing was lower in CSDS animals for the 2.5 min (W=538, p=0.002), 5 min (W=576, p=0.0003), and 7.5 min (W=499, p=0.012), but not the 10 min (W=456, p=0.06). Kruskall-Wallis test: 2.5 min: p=0.002, 5 min: p=0.0003, 7.5 min: p=0.012, and 10 min: p=0.06. C) B-wall-climbing is lower in stressed animals. Wilcoxon test: W=540, p=0.0004. D) Wilcoxon posthoc analysis revealed that B-wall-climbing was lower in stressed animals for the 2.5 min (W=441, p=0.03), the 5 min (W=435, p=0.03), and the 7.5 min (W=506, p=0.002), but not the 10 min (W=393, p=0.37). Kruskall-Wallis test: 2.5 min: p=0.03, 5 min: p=0.03, 7.5 min: p=0.002, and 10 min: p=0.37. E) Side-by-side is lower in CSDS animals. Wilcoxon test: W=522.5, p=0.0023. F) Wilcoxon posthoc analysis revealed that Side-by-side was lower in CSDS animals for the 2.5 min (W=581, p=5.48e-5), the 5 min (W=521.5, p=0.003), and the 10 min (W=491.5, p=0.02), but not the 7.5 min (W=405, p=0.32). Kruskall-Wallis test: 2.5 min: p=5.28e-5, 5 min: p=0.003, 7.5 min: p=0.32, and 10 min: p=0.02. G) Side-reverse-side is not altered between conditions. Wilcoxon test: W=365, p=0.81. H) Wilcoxon posthoc analysis revealed that Side-reverse-side was lower in CSDS animals for the 2.5 min time bin (W=628, p=3.36e-6), but not the 5-, 7.5-, and 10 min time bins (W=337.5, p=1; W=292.5, p=0.60; W=351, p=1, respectively). Kruskall-Wallis test: 2.5 min: p=3.21e-6, 5 min: p=1, 7.5 min: p=0.60, and 10 min: p=1. I) Nose-to-nose is not altered between conditions. Wilcoxon test: W=326, p=0.67. J) No further significant differences were observed in the Nose-to-nose time bins. The timeline and bar graphs are presented as mean ± standard error of the mean and all individual samples as points. N=26 for NS and N=27 for CSDS in panels A-J. Source data are provided as a Source Data file.

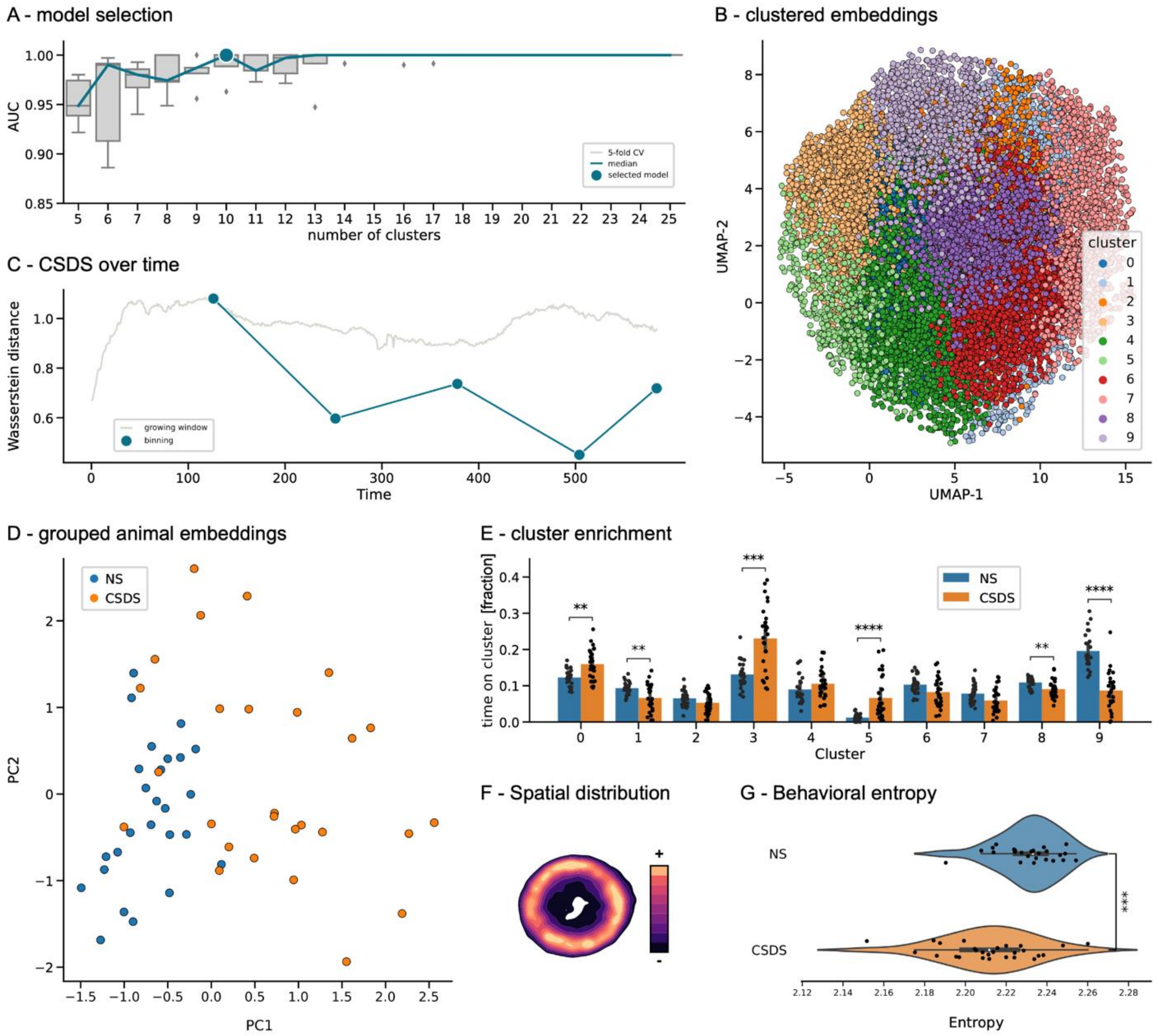

Supplemental Figure 5. Multi-animal unsupervised analyses identify different two-mice behavioral patterns between arenas containing stressed and non-stressed mice during the SI task. A) Cluster selection pipeline results, reporting the area under the ROC curve from a logistic regression classifier discriminating between conditions. A 10-component solution (from a range between 5 and 25) was selected as optimal in a 5-fold (N=5) cross-validation loop (see methods for details). B) Embeddings by time point obtained using DeepOF's unsupervised pipeline. Different colors correspond to different clusters. Dimensionality was further reduced from the original 8-dimensional embeddings using UMAP for visualization purposes. C) Optimal binning of the videos was obtained as the Wasserstein distance between the global animal embeddings of both conditions across a growing window, between the first 10 to 600 seconds for each video at one-second intervals (grey curve). Higher values correspond to larger behavioral differences across conditions. A maximum was observed at 124 seconds, close to the 126 seconds obtained with the single-animal embeddings, and to the stipulated 150 seconds selected based on the SA task literature. The dark green curve depicts the Wasserstein distance across all subsequent non-overlapping bins with optimal length. The decay observed across time is consistent with the hypothesized arousal period in the CSDS cohort, which can be detected also embedding the two-mice system as a whole. D) Representation of the global animal embeddings for the optimally discriminant bin

(124 seconds) per experimental video colored by condition (see methods for details). E) Cluster enrichment per experimental condition (N=26 for NS and N=27 for CSDS) in the first optimal bin (first 124 seconds). Reported statistics correspond to a 2-way Mann-Whitney U non-parametric test corrected for multiple testing using the Benjamini-Hochberg method across both clusters and bins (significant differences observed in clusters 0: U=1.7e+2, p=1.2e-3, 1: U=4.9e+2, p=8.5e-3, 3: U=1.4e+2, p=1.4e-4, 5: U=8.4e+1, p=2.1e-6, 8: U=5.3e+2, p=1.2e-3, 9: U=6.7e+2, p=1.4e-8). Bar graphs represent mean ± standard deviation of the time proportion spent on each cluster. F) Example heatmap depicting spatial distribution across all experiments (in both conditions) for all clusters. Specific heatmaps for all individual clusters are available in supplemental figure 13). G) Behavioral entropy scores per condition. NS animals show a significantly higher entropy than CSDS animals, which can be attributed to a less predictable exploration of the behavioral space (U=5.44e+2, p=6.15e-4, N=26 for NS and N=27 for CSDS). Moreover, and in accordance with these results, behavioral entropy shows a significant negative correlation with the presented stress physiology Z-score (supplemental figure 15B). Source data are provided as a Source Data file. Box plots in panels A and G show the median and the inter-quartile range. Whiskers show the full range, excluding outliers as a function of the inter-quartile range.

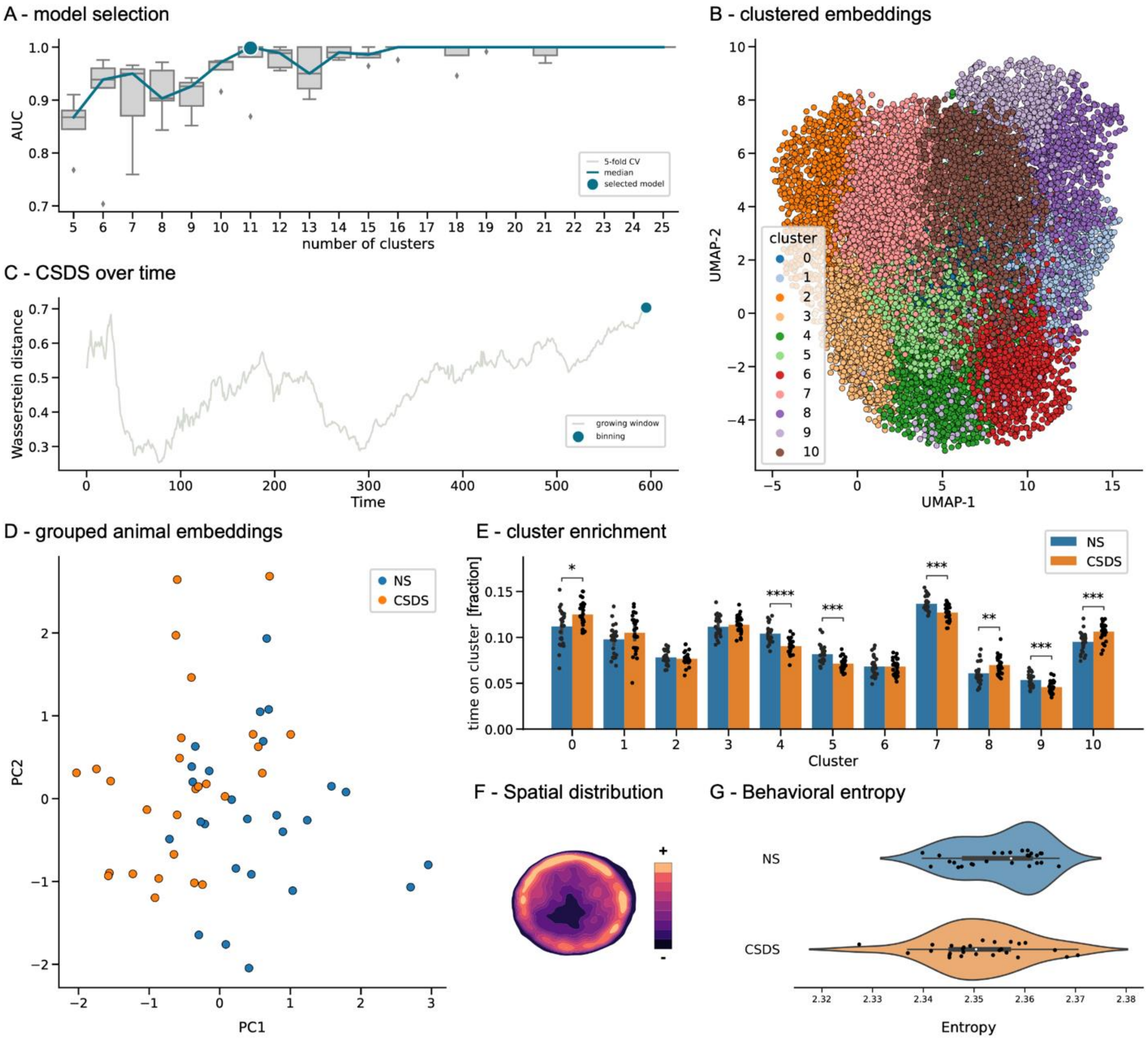

Supplemental Figure 6. Single-animal unsupervised analyses identify different behavioral patterns between stressed and non-stressed mice during the OF task. A) Cluster selection pipeline results, reporting the area under the ROC curve from a logistic regression classifier discriminating between conditions. An 11-component solution (from a range between 5 and 25) was selected as optimal in a 5-fold (N=5) cross-validation loop (see methods for details). B) Embeddings by time point obtained using DeepOF's unsupervised pipeline. Different colors correspond to different clusters. Dimensionality was further reduced from the original 8-dimensional embeddings using UMAP for visualization purposes. C) Optimal binning of the videos was obtained as the Wasserstein distance between the global animal embeddings of both conditions across a growing window, between the first 10 to 600 seconds for each video at one-second intervals (grey curve). Higher values correspond to larger behavioral differences across conditions. A maximum was observed at 595 seconds (green dot), which is consistent with the hypothesized lack of an arousal period in the CSDS cohort in an open field setting with no conspecific. D) Representation of the global animal embeddings for the optimally discriminant bin (595 seconds) per experimental video colored by condition (see methods for details). E) Cluster enrichment per

experimental condition (N=26 for NS and N=27 for CSDS) in the first optimal bin (first 595 seconds). Reported statistics correspond to a 2-way Mann-Whitney U non-parametric test corrected for multiple testing using the Benjamini-Hochberg method across both clusters and bins (significant differences observed in clusters 0: U=2.2e+2, p=2.02e-2, 4: U=6.1e+2, p=5.7e-6, 5: U=5.7e+2, p=1.3e-4, 7: U=5.4e+1, p=9.9e-4, 8: U=1.8e+2, p=2.3e-3, 9: U=5.5e+2, p=3.7e-4, and 10: U=1.5e+2, p=2.6e-4. Bar graphs represent mean ± standard deviation of the time proportion spent on each cluster. F) Example heatmap depicting spatial distribution across all experiments (in both conditions) for all clusters. Specific heatmaps for all individual clusters are available in supplemental figure 14). G) Behavioral entropy scores per condition. No significant differences are detected between conditions (U=4.44e+2, p=9.98e-2, N=26 for NS and N=27 for CSDS). Moreover, and in accordance with these results, no significant correlation with the presented stress physiology Z-score was found (supplemental figure 15C). Source data are provided as a Source Data file. Box plots in panels A and G show the median and the inter-quartile range. Whiskers show the full range, excluding outliers as a function of the inter-quartile range.

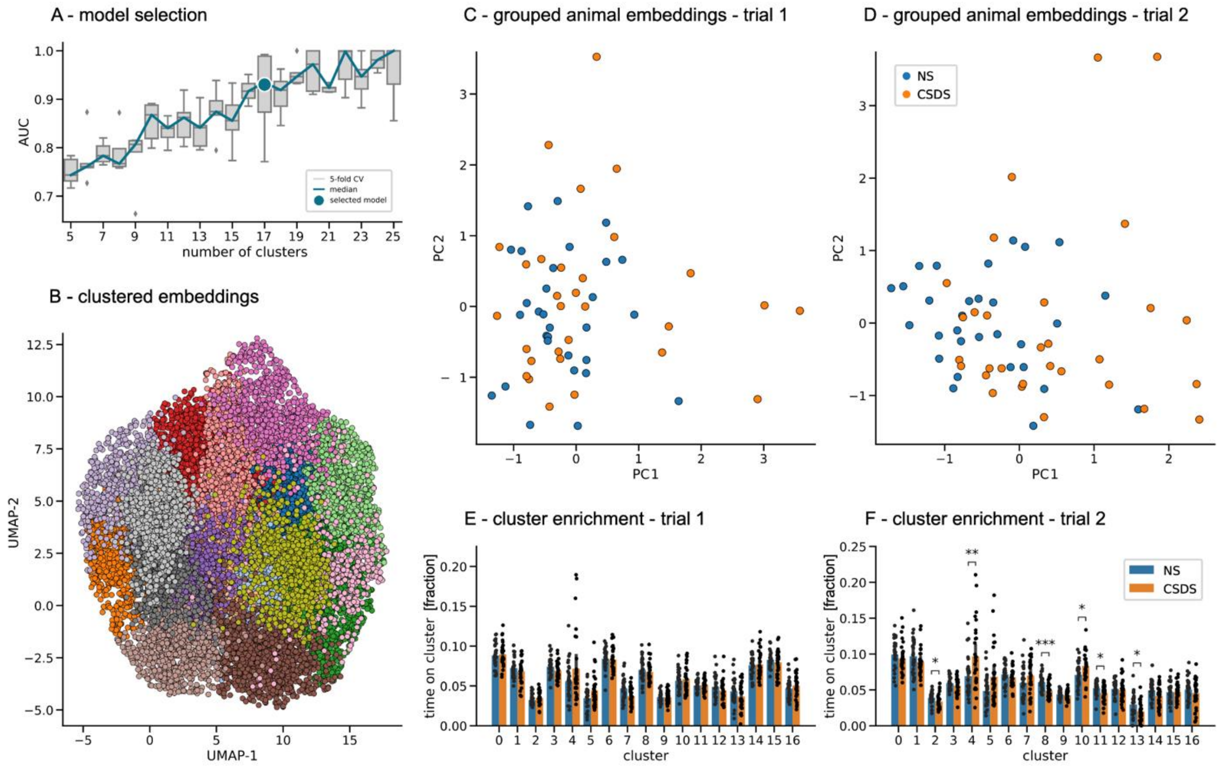

Supplemental Figure 7. Single-animal unsupervised analyses identify mild behavioral differences between stressed and non-stressed mice during the SA task. A) Cluster selection pipeline results. Models ranging from 5 to 25 clusters were trained in a 5-fold (N=5) cross-validation loop using data from both trials together. Area under the ROC curve from a logistic regression classifier discriminating between conditions on the global animal embeddings representing the differential population of each cluster across trials is reported is reported. A 17-component solution was selected as the smallest whose median performance deviated less than one standard deviation from the maximum reached median across all clusters. Boxes in the box plots show the median performance and the inter-quartile range of the data. Whiskers show the full range of the data, excluding outliers as a function of the inter-quartile range. B) Embeddings by time point obtained using DeepOF's unsupervised pipeline. Different colors correspond to different clusters. Dimensionality was further reduced from the original 8-dimensional embeddings using UMAP for visualization purposes. C-D) Representation of the global animal embeddings per experimental video colored by condition, for SA trials one (without conspecific in the cage) and two (with conspecific in the cage). In panel C, as expected, the distributions are further apart. E-F) Cluster enrichment per experimental condition for both SA trials (N=30 for NS and N=30 for CSDS). As expected, trial one shows no significant differences, whereas trial two yields six significantly differentially expressed clusters. Reported statistics correspond to a 2-way Mann-Whitney U non-parametric test corrected for multiple testing using the Benjamini-Hochberg method across both clusters (significant differences for trial two observed in clusters 2: U=6.1e+2, p=1.4e-2, 4: U=2.6e+2, p=7.3e-6, 8: U=7.01e+2, p=2.1e-4, 10: U=2.8e+2, p=1.4e-2, 11: U=6.1e+2, p=1.7e-2, and 13: U=6.1e+2, p=1.8e-2. Bar graphs represent mean ± standard deviation of the time proportion spent on each cluster. Source data are provided as a Source Data file.

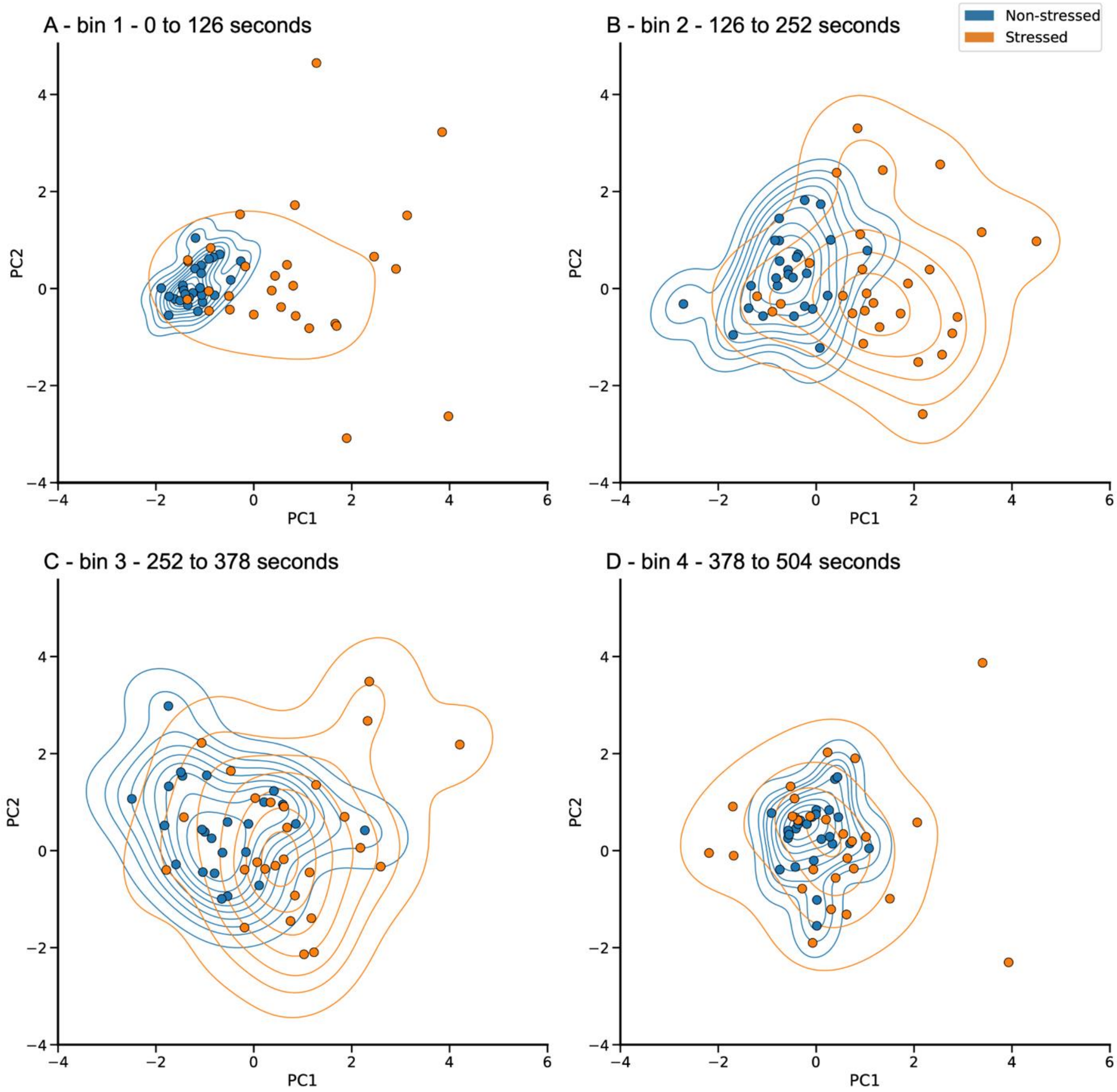

Supplemental figure 8. Global single-animal embeddings across non-overlapping time bins in the SI dataset. A-D) 10-dimensional global single-animal embeddings were obtained as the time proportion spent on each of the 10 clusters in the selected model for the single-animal SI task. Panels A to D show how the distributions matching NS and CSDS animals get closer and closer across non-overlapping consecutive time bins (as quantified using Wasserstein distance in the first four points shown in dark green in figure 6B). The last bin was excluded for visualization purposes. Source data are provided as a Source Data file.

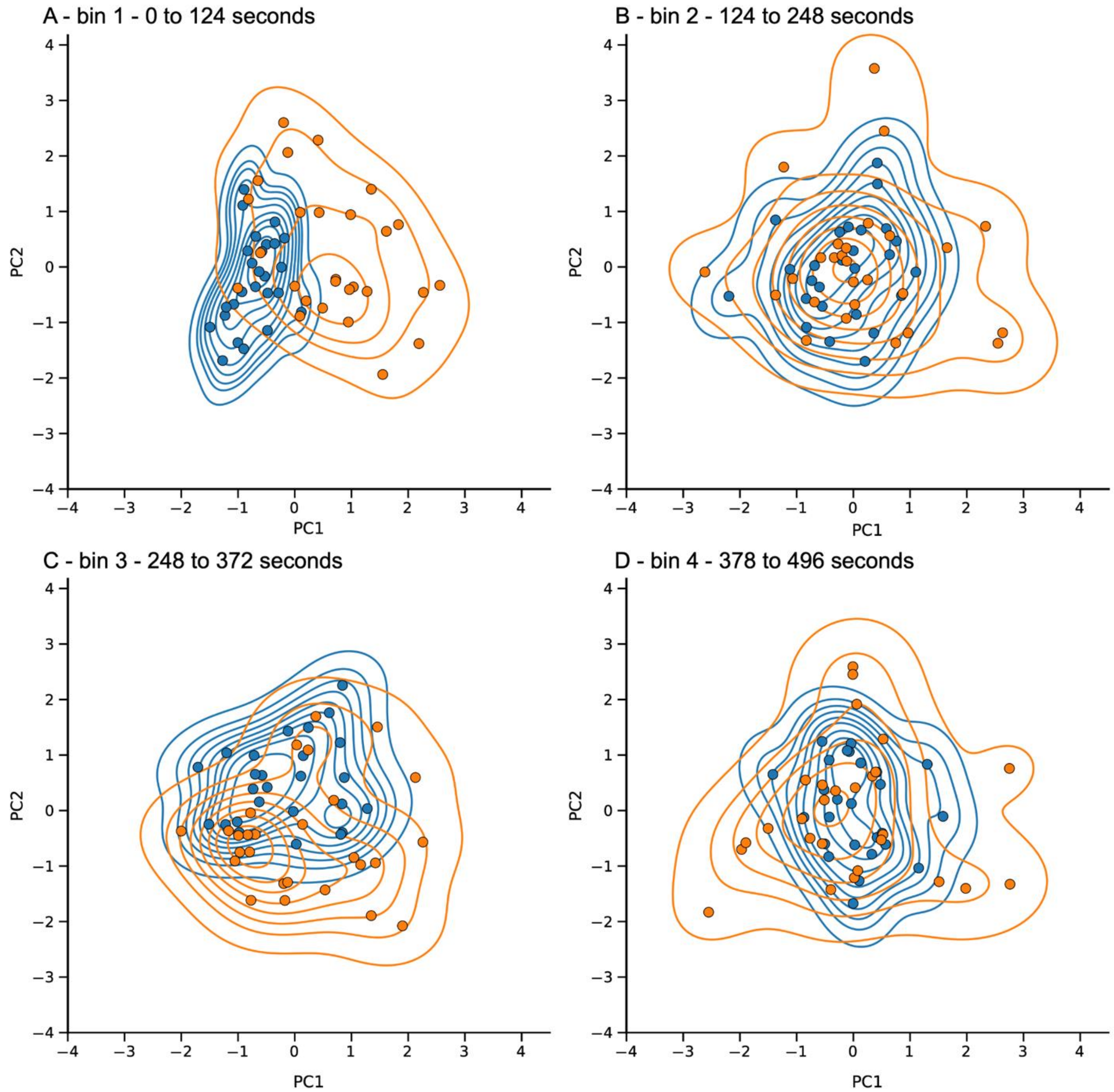

Supplemental figure 9. Global multi-animal embeddings across non-overlapping time bins in the SI dataset. A-D) 10-dimensional global single-animal embeddings were obtained as the time proportion spent on each of the 10 clusters in the selected model for the multi-animal SI task. Panels A to D show how the distributions matching NS and CSDS animals get closer across non-overlapping consecutive time bins (as quantified using Wasserstein distance in the first four points shown in dark green in supplemental figure 9B). The last bin was excluded for visualization purposes. Source data are provided as a Source Data file.

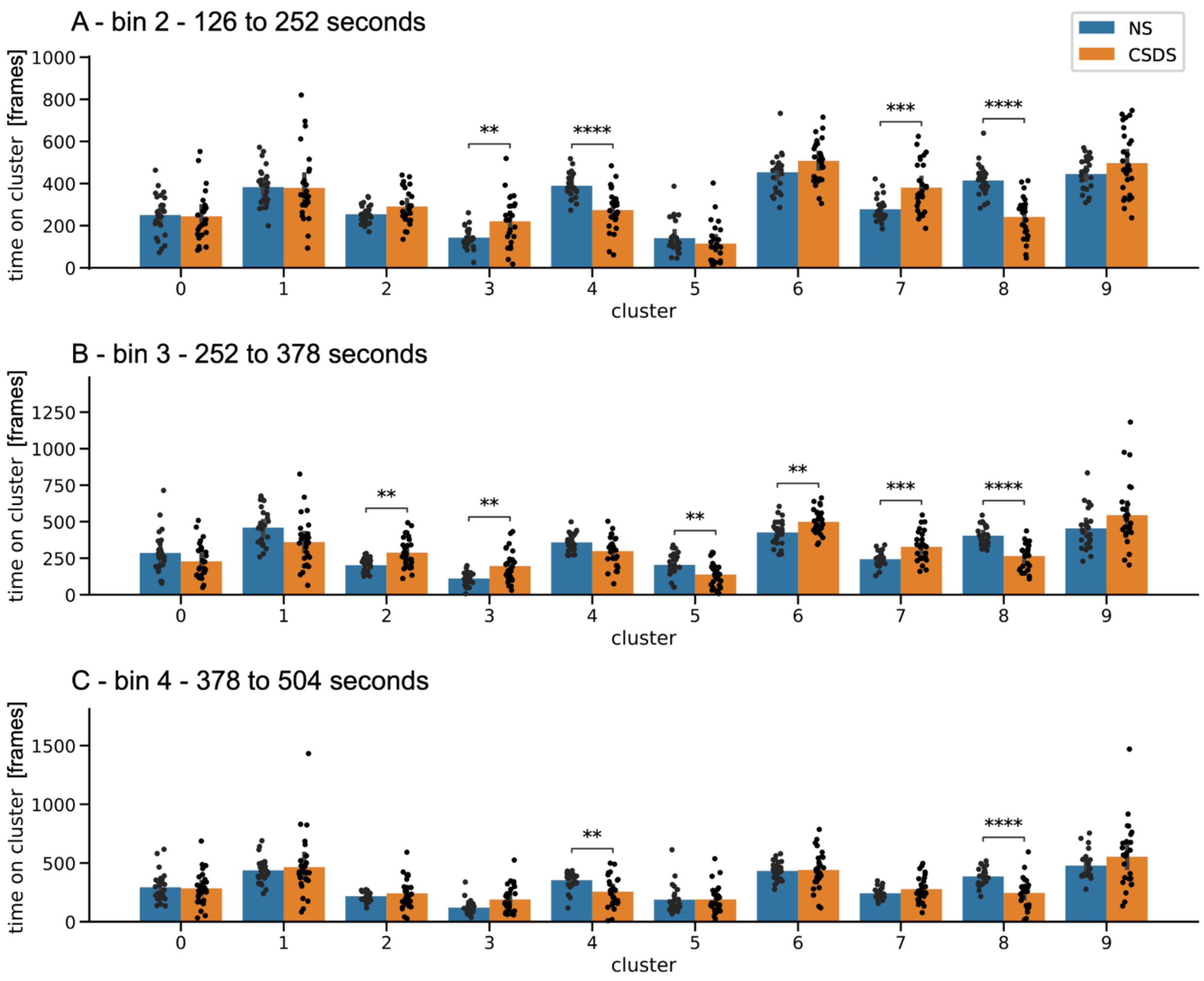

Supplemental figure 10. Cluster enrichment per experimental condition in the second to fourth optimal bins for the single-animal embeddings on the SI task. Reported statistics correspond to a 2-way Mann-Whitney U non-parametric test corrected for multiple testing using the Benjamini-Hochberg method across both clusters and bins. In all cases, N=26 for NS and N=27 for CSDS. A) Second bin (126 to 252 seconds). Significant differences observed in clusters 3: U=1.9e+2, p=6.3e-10, 4: U=5.9e+2, p=1.4e-5, 7: U=1.6e+2, p=6.9e-4, and 8: U=6.55e+2, p=6.3e-8 B) Third bin (252 to 378 seconds). Significant differences observed in clusters 2: U=1.8e+2, p=1.8e-3, 3: U=1.7e+2, p=1.2e-3, 5: U=4.9e+2, p=8.5e-3, 6: U=1.9e+2, p=7.01e-3, 7: U=1.7e+2, p=9.6e-4, and 8: U=6.3e+2, p=6.6e-7. C) Fourth bin (378 to 504 seconds). Significant differences observed in clusters 4: U=5.2e+2, p=2.5e-5, and 8: U=6.02e+2, p=6.5e-6. Bar graphs represent mean ± standard deviation of the time proportion spent on each cluster. Source data are provided as a Source Data file.

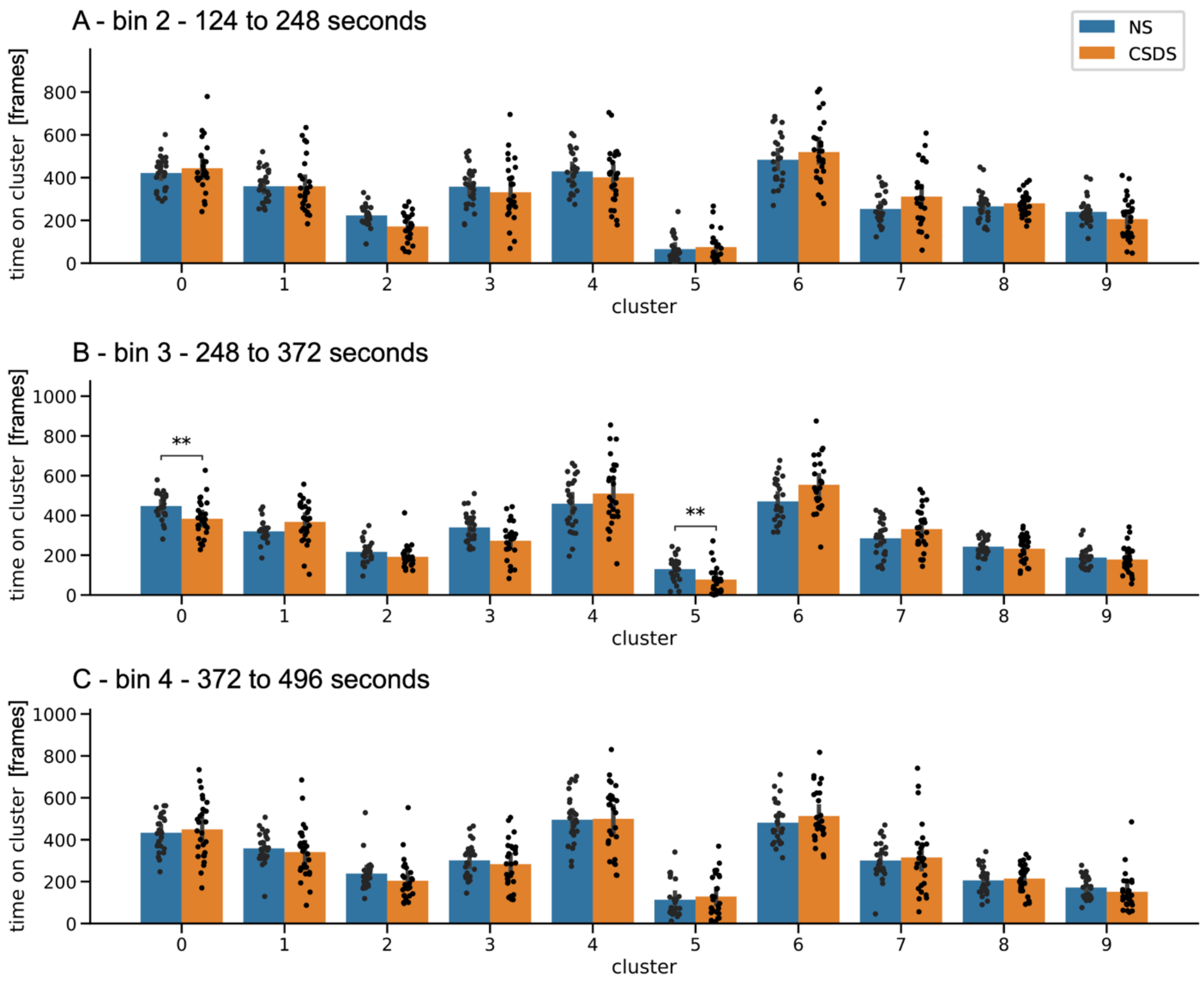

Supplemental figure 11. Cluster enrichment per experimental condition in the second to fourth optimal bins reported for the multi-animal embeddings on the SI task. Reported statistics correspond to a 2-way Mann-Whitney U non-parametric test corrected for multiple testing using the Benjamini-Hochberg method across both clusters and bins. In all cases, N=26 for NS and N=27 for CSDS. A) Second bin (124 to 248 seconds). No significant differences observed. B) Third bin (248 to 372 seconds). Significant differences were observed in clusters 0: U=5.2e+2, p=3.3e-3, and 5: U=5.3e+2, p=1.6e-3. C) Fourth bin (372 to 496 seconds). No significant differences were observed. Bar graphs represent mean ± standard deviation of the time proportion spent on each cluster. Source data are provided as a Source Data file.

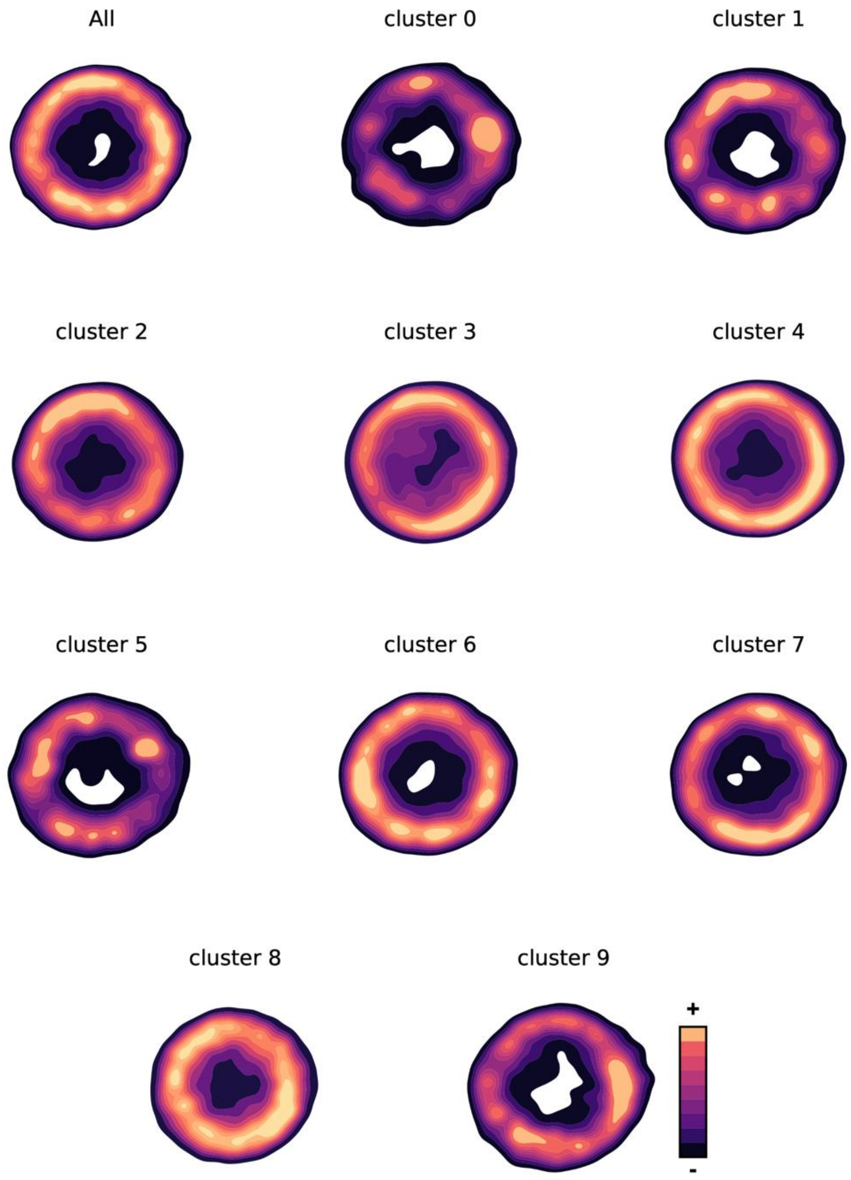

Supplemental figure 12. Spatial distribution of clusters obtained using single-animal embeddings in the SI task. Heatmaps include full trajectories of all experiments in both conditions, filtering time points belonging to each obtained cluster, and without filtering (labelled as "all"). White background indicates null population of the area. All clusters enriched in CSDS show lower occupation of the center of the arena than those enriched in NS animals.

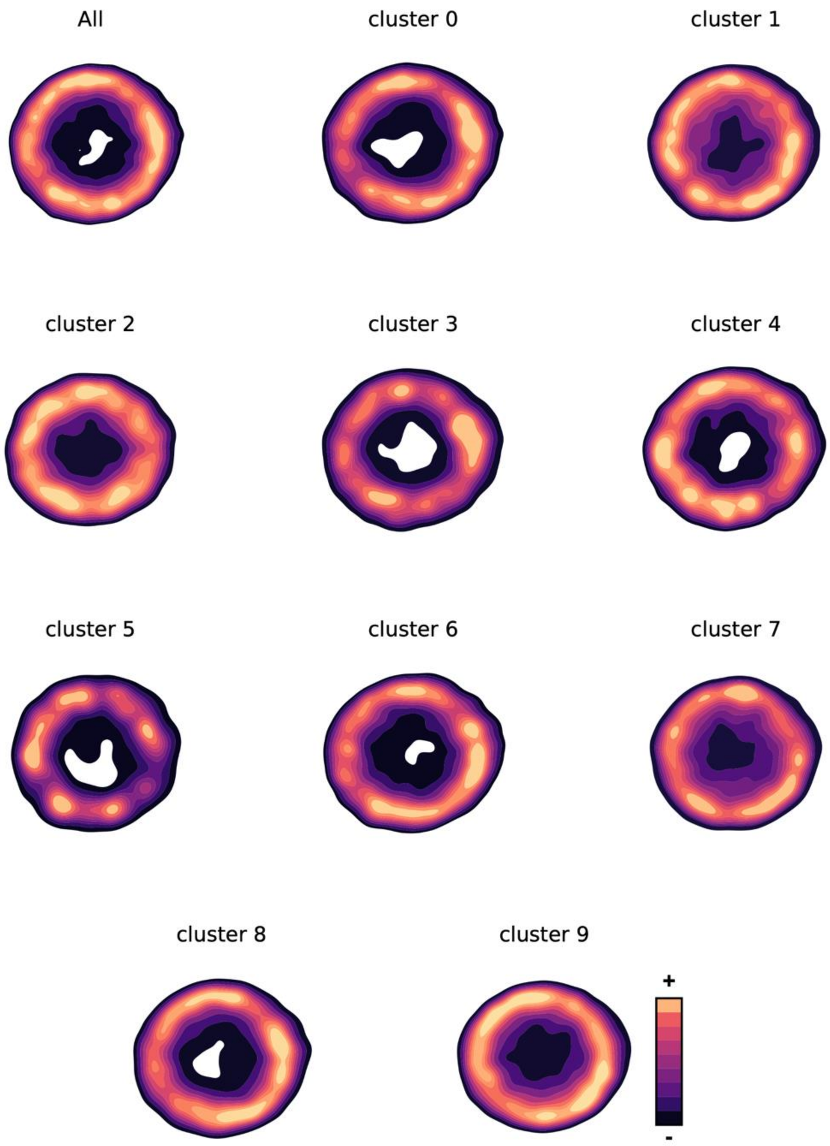

Supplemental figure 13. Spatial distribution of clusters obtained using multi-animal embeddings in the SI task. Heatmaps include full trajectories of all experiments in both conditions, filtering time points belonging to each obtained cluster, and without filtering (labelled as "all"). White background indicates null population of the area. All clusters enriched in CSDS show lower occupation of the center of the arena than those enriched in NS animals.

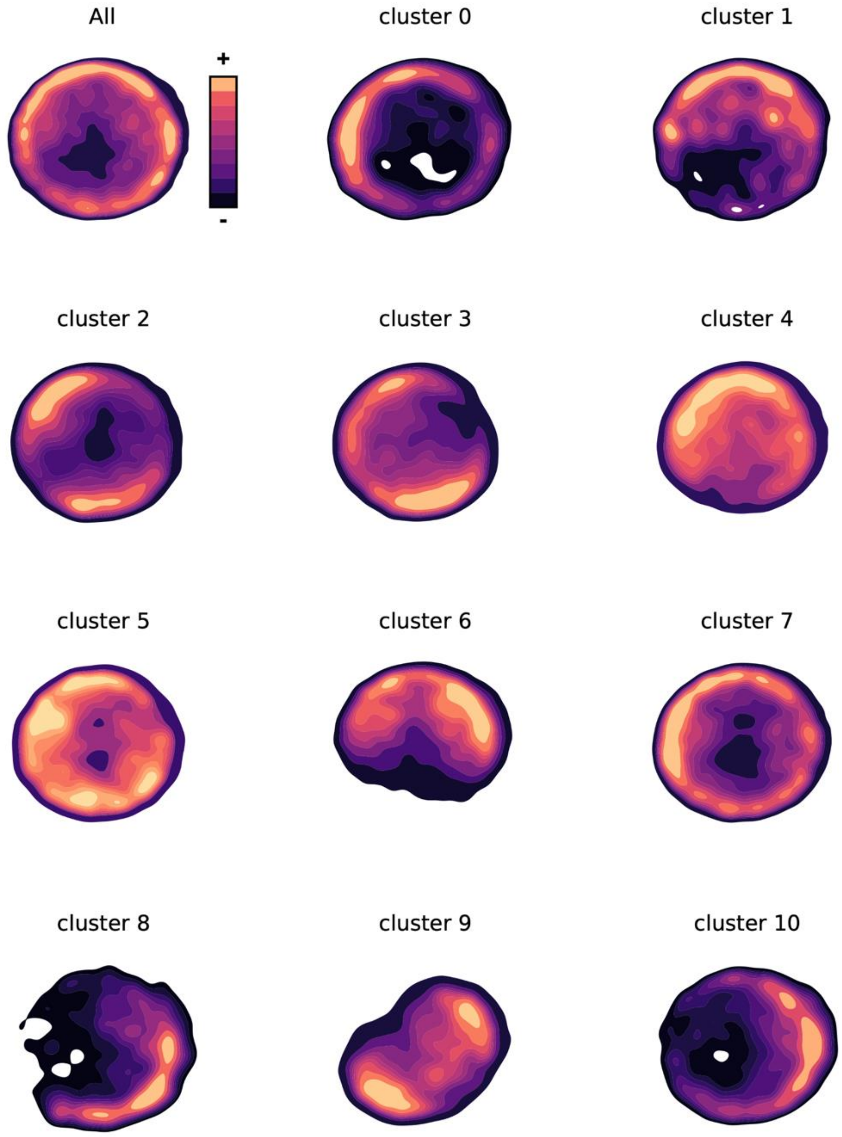

Supplemental figure 14. Spatial distribution of clusters obtained in the OF task. Heatmaps include full trajectories of all experiments in both conditions, filtering time points belonging to each obtained cluster, and without filtering (labelled as "all"). White background indicates null population of the area. All clusters enriched in CSDS show lower occupation of the center of the arena than those enriched in NS animals.

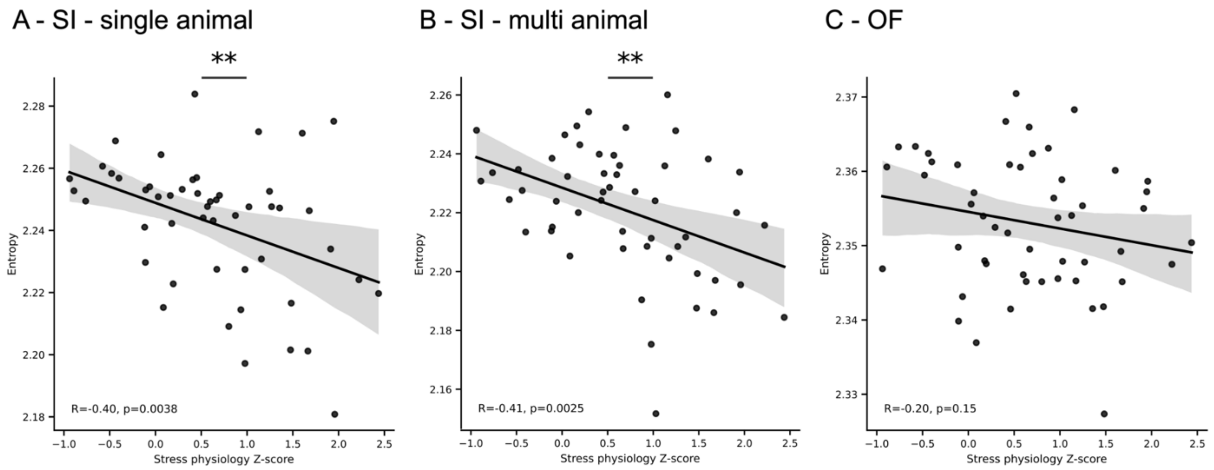

Supplemental Figure 15. Correlation between behavioral entropy and stress physiology Z-score. A) Behavioral entropy of the cluster space obtained with single animal embeddings during the social interaction (SI) task shows a significant negative Pearson correlation with the stress physiology Z-score (R=-0.40, p=3.8e-3, N=53). Error bands represent the 95% confidence band around the mean of the linear model. B) Behavioral entropy of the cluster space obtained with multi-animal embeddings during the social interaction (SI) task shows a significant negative Pearson correlation with the stress physiology Z-score (R=-0.41, p=2.5e-3, N=53). Error bands represent the 95% confidence band around the mean of the linear model C) Behavioral entropy of the cluster space obtained during the open field (OF) task shows no significant Pearson correlation with the stress physiology Z-score (R=-0.20, p=0.15, N=53). Error bands represent the 95% confidence band around the mean of the linear model. All three tests are two-sided. Source data are provided as a Source Data file.

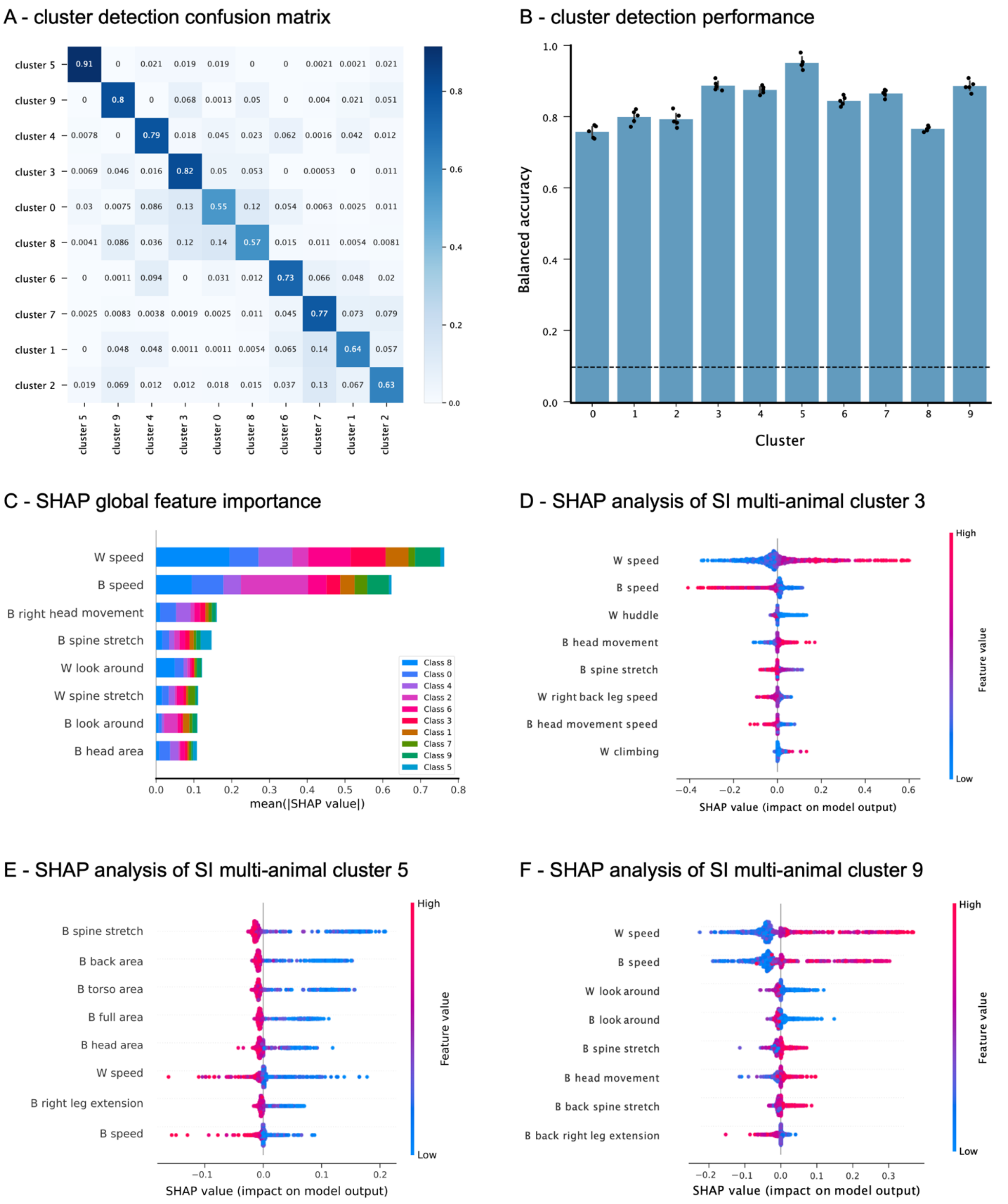

Supplemental Figure 16. SHAP analysis of unsupervised cluster assignments in the multi-animal social interaction task. Gradient boosting machines were trained to map from a predefined set of time series statistics (including body part speeds, distances, distance speeds, areas, area speeds, and supervised annotations for each of the two animals and their interaction) to the previously obtained cluster assignments. A) Confusion matrix obtained from the trained gradient boosting machine classifying

between clusters. Aggregated performance over the validation folds of a 5-fold cross-validation is shown. B) Validation performance per cluster across a 5-fold (N=5) cross-validation loop. Balanced accuracy was used to correct for cluster assignment imbalance. The dashed line marks the expected performance due to chance, considering all outputs. Bars show mean ± 95% confidence interval. C) Overall feature importance for the multi-output classifier using SHAP. Features in the y-axis are sorted by overall absolute SHAP values across clusters. Classes on the bars are sorted by overall absolute SHAP values across features. D-F) Bee swarm plots for the three most differentially expressed clusters between NS and CSDS mice (3, 5, and 9), identified with the unsupervised DeepOF pipeline on the SI experiments using single-animal embeddings. The depicted plots display the first 8 most important features for each classifier, in terms of the mean absolute value of the SHAP values. Source data are provided as a Source Data file.

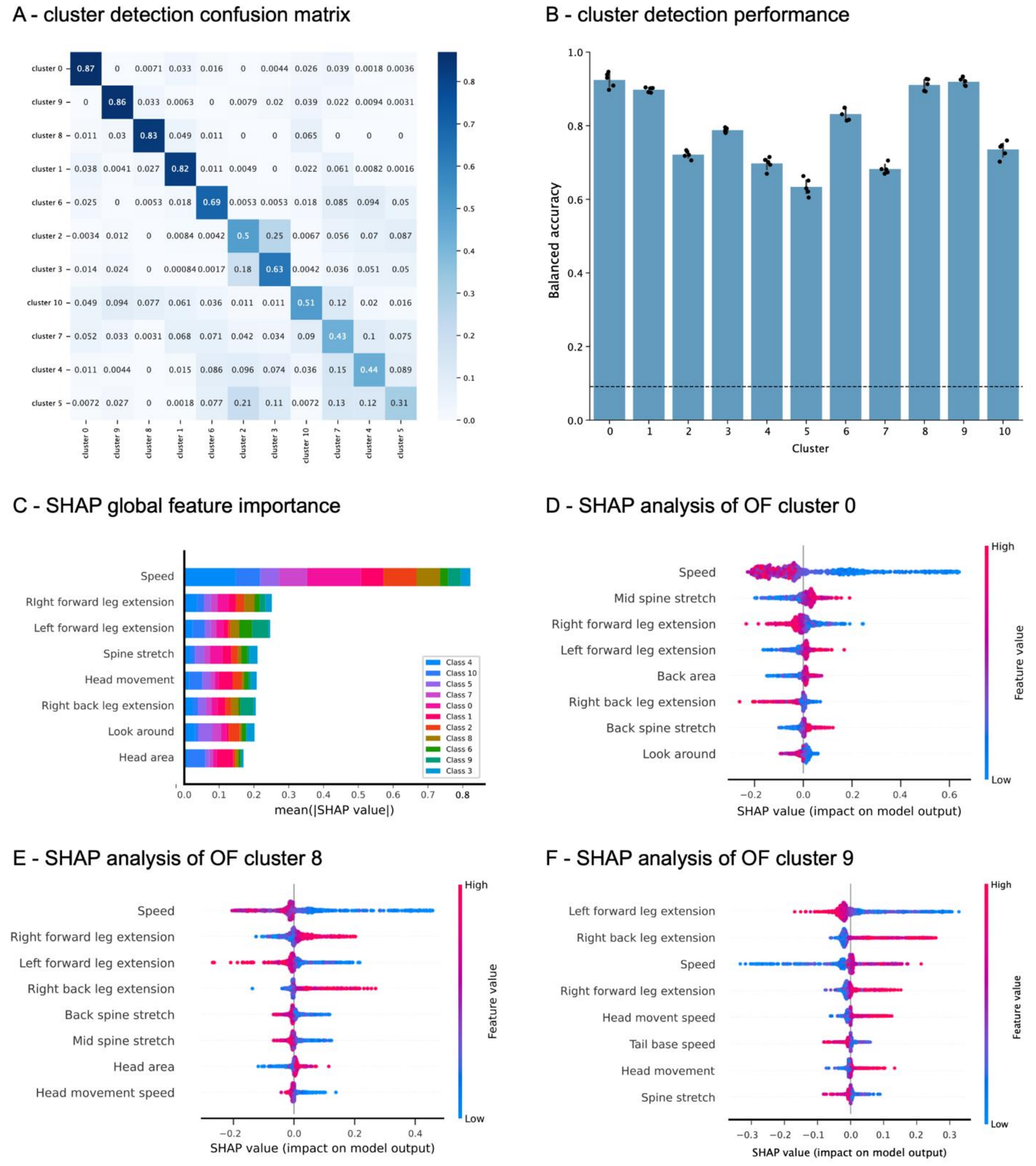

Supplemental Figure 17. SHAP analysis of unsupervised cluster assignments in the open field task. Gradient boosting machines were trained to map from a predefined set of time series statistics (including body part speeds, distances, distance speeds, areas, area speeds, and supervised annotations) to the previously obtained cluster assignments. A) Confusion matrix obtained from the trained gradient boosting machine classifying between clusters. Aggregated performance over the validation folds of a 5-fold cross-validation is shown. B) Validation performance per cluster across a 5-fold (N=5) cross-validation loop. Balanced accuracy was used to correct for cluster assignment imbalance. The dashed line marks the

expected performance due to chance, considering all outputs. Bars show mean ± 95% confidence interval. C) Overall feature importance for the multi-output classifier using SHAP. Features in the y-axis are sorted by overall absolute SHAP values across clusters. Classes on the bars are sorted by overall absolute SHAP values across features. D-F) Bee swarm plots for the three most differentially expressed clusters between NS and CSDS mice (4, 9, and 10), identified with the unsupervised DeepOF pipeline on the SI experiments using single-animal embeddings. The depicted plots display the first 8 most important features for each classifier, in terms of the mean absolute value of the SHAP values. Source data are provided as a Source Data file.
